# Genetic Aberrations Associated with Photodynamic Therapy in Colorectal Cancer Cells

**DOI:** 10.3390/ijms20133254

**Published:** 2019-07-02

**Authors:** Heidi Abrahamse, Nicolette Nadene Houreld

**Affiliations:** Laser Research Centre, Faculty of Health Sciences, University of Johannesburg, Johannesburg 2028, South Africa

**Keywords:** photodynamic therapy, colorectal cancer, zinc phthalocyanine, apoptotic pathway

## Abstract

Photodynamic therapy (PDT) is a cancer treatment modality that utilizes three components: light (λ 650–750 nm), a photosensitizer (PS) and molecular oxygen, which upon activation renders the modality effective. Colorectal cancer has one of the highest incident rates as well as a high mortality rate worldwide. In this study, a zinc (Zn) metal-based phthalocyanine (ZnPcSmix) PS was used to determine its efficacy for the treatment of colon adenocarcinoma cells (DLD-1 and Caco-2). Photoactivation of the PS was achieved by laser irradiation at a wavelength of 680 nm. Dose responses were performed to establish optimal PS concentration and irradiation fluence. A working combination of 20 µM ZnPcSmix and 5 J/cm^2^ was used. Biochemical responses were determined after 1 or 24 h incubation post-treatment. Since ZnPcSmix is localized in lysosomes and mitochondria, mitochondrial destabilization analysis was performed monitoring mitochondrial membrane potential (MMP). Cytosolic acidification was determined measuring hydrogen peroxide (H_2_O_2_) levels in the cytoplasm. Having established apoptotic cell death induction, an apoptosis PCR array was performed to establish the apoptotic mechanism. In DLD-1 cells, expression of genes included 3 up-regulated and 20 down-regulated genes while in Caco-2 cells, there were 16 up-regulated and 22 down-regulated genes. In both cell lines, in up-regulated genes, there was a combination of pro- and anti-apoptotic genes that were significantly expressed. Gene expression results showed that more tumorigenic cells (DLD-1) went through apoptosis; however, they exhibit increased risk of resistance and recurrence, while less tumorigenic Caco-2 cells responded better to PDT, thus being suggestive of a better prognosis post-PDT treatment. In addition, the possible apoptotic mechanisms of cell death were deduced based on the genetic expression profiling of regulatory apoptotic inducing factors.

## 1. Introduction

Colorectal cancer is highly metastatic due to amplified migration, proliferation and adhesion rate. The disease is also difficult to treat due to the prevalent recurrence rate. Cancer progresses as a result of the cells’ failure to undergo genetically regulated apoptosis. The apoptotic cell death pathway is a preferred mode of cell death as it plays an important role in the regulation of homeostasis. Adjuvant chemotherapy, radiotherapy, surgical removal or a combination of the latter treatments for the disease yields successful removal of the tumor mass, but does not improve patient morbidity and survival or reduce the recurrence rate. Moreover, the success of the latter treatments is limited by the progression stage of the cancer [1].

PDT is a minimally invasive therapy that has a better potential compared to the aforementioned therapies. It does not induce resistance, can be applied repeatedly without compromising the immune response, and can be used in conjunction with other cancer therapies. Phthalocyanines are among the second-generation PSs. They have attractive photochemical properties for use in PDT, and are therefore worth investigating for colorectal cancer treatment. In South Africa the treatment is not approved or even reached the clinical trials stage due to insufficient research data. It is, therefore, essential to pursue fundamental research as it may provide guidance to oncologists interested in alternative cancer treatment in the future.

The localization site of a PS is indicative of where the initial cellular damage will occur [2,3]. Kessel and Luo (1998) indicated that sub-cellular localization of the PS is a primary determinant of the cell death mechanism in PDT [4]. Reactive Oxygen Species (ROS) such as hydroxyl radicals (·OH) and hydrogen peroxide (H_2_O_2_) are normally generated in small amounts in cellular organisms due to aerobic metabolism [5]. Oxidative stress caused by increased ROS concentration cause cellular damage, and ROS induction is implicated in apoptosis [6]. Oxidative stress caused by photodynamic reactions or ROS production can directly disrupt the organelle membrane/s by peroxidation of membrane lipids [7].

It has become conventional wisdom that photodamage will occur at the primary site of PS localization because photogenerated ROS such as ^1^O_2_ are short-lived with a partial diffusion pathway into biological systems [8]. Moreover, determination of these components would make it feasible to determine or suggest which cell death mode may herald post-PDT. Release of lysosomal contents can trigger apoptosis or apoptosis-like cell death. Massive oxidative stress leads to an increase in H_2_O_2_ in the cytosol, which is indicative of a decrease in pH gradient and proton pump failure that results in damage to the plasma membrane. Ahmad et al. (2004) suggested that H_2_O_2_ production could lead to induction of Bax translocation and subsequently result in cytochrome C release [9]. This mechanism depletes the mitochondrial electron transport chain with the consequential leakage of electrons to form O^2−^, which is dismutated by mitochondrial superoxide dismutase (SOD) to cytotoxic H_2_O_2_ leading to cytosolic acidification.

Loss of mitochondrial membrane potential (MMP) is recognized as a cell death signal, as it signifies loss of normal mitochondrial function, which as a result mitochondrial contents leaking into the cytosolic space and triggering other elements to proceed with cell death. In addition, regardless of caspase activation, loss of MMP will result in the release of caspase-activating molecules, caspase-independent death effectors and metabolic failure in mitochondria [10]. Consequently, caspase activation is linked with mitochondrial depolarization since it is activated by mitochondrial components in the cytosol, predominantly by cytochrome C [11].

Apoptosis is used synonymously with programmed cell death (PCD) and is known to be an energy-requiring mechanism that is tightly controlled biochemically and genetically and is divided into two pathways: the extrinsic or death receptor pathway and the intrinsic pathway, also known as the mitochondrial pathway. Both pathways are highly regulated and involve an extremely intricate play of several factors that activate either. PDT and the use of specific PSs has proven to be highly specific in how it effectively eradicates cancer cells. A PS that may prove highly efficient for one type of cancer may prove less effective in another. The underlying mechanism of cell death induction must be established to determine the efficacy of PDT using specific PSs.

## 2. Results

### 2.1. Cytosolic Acidification

**Figure 1:**
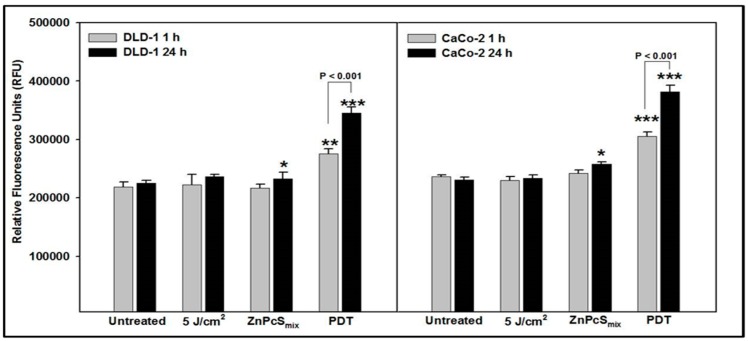
Hydrogen peroxide (H_2_O_2_) was determined after 1 and 24 h post-treatment and relative fluorescence units were measured (530Ex/590Em). Significant differences as compared to untreated control cells is shown as * *p* < 0.05, ** *p* < 0.01 and *** *p* < 0.001. There were significantly increased H_2_O_2_ levels in PDT treated DLD-1 and Caco-2 cells after both 1 and 24 h incubation.

After 1 h incubation, irradiated (5 J/cm^2^) and ZnPcSmix treated DLD-1 cells showed no significant difference in H_2_O_2_ levels in comparison to untreated control cells (Figure 1). However, PDT treated DLD-1 cells showed a significant difference in comparison to their untreated control cells (*p* < 0.01). Irradiated (5 J/cm^2^) DLD-1 cells were not significantly different when compared to the same cells treated with ZnPcSmix alone or PDT treated cells. There was a significant increase in H_2_O_2_ levels in PDT treated DLD-1 cells compared to those treated with ZnPcSmix alone (*p* < 0.001). After 24 h incubation, DLD-1 cells treated with ZnPcSmix alone as well as PDT treated cells showed a significant increase in H_2_O_2_ levels compared to the untreated control cells (*p* < 0.05 and *p* < 0.001, respectively). PDT treated DLD-1 cells showed a significant increase as compared to both irradiated (5 J/cm^2^) and ZnPcSmix treated cells (*p* < 0.001 and *p* < 0.01, respectively). When incubation times were compared, H_2_O_2_ levels in PDT treated DLD-1 cells was significantly increased after 24 h (*p* < 0.001). Analysis of Caco-2 cells (Figure 1) showed that after 1 h incubation, irradiated (5 J/cm^2^) and ZnPcSmix treated cells showed no significant difference in H_2_O_2_ levels compared to untreated control cells, while PDT treated DLD-1 cells showed a significant increase (*p* < 0.001). Comparison of irradiated (5 J/cm^2^) and ZnPcSmix treated Caco-2 cells had significantly decreased H_2_O_2_ levels compared to PDT treated cells (*p* < 0.001). Twenty-four hours post-treatment, Caco-2 cells treated with ZnPcSmix alone as well as PDT treated cells showed a significant increase in H_2_O_2_ levels as compared to untreated control cells (*p* < 0.05 and *p* < 0.001, respectively).

Irradiated (5 J/cm^2^) Caco-2 cells showed significantly less H_2_O_2_ compared to ZnPcSmix alone (*p* < 0.01) and PDT treated cells (*p* < 0.001), and cells treated with ZnPcSmix alone resulted in significantly less H_2_O_2_ than PDT treated Caco-2 cells (*p* < 0.001). When incubation times were compared, H_2_O_2_ levels in PDT treated Caco-2 cells was significantly increased after 24 h (*p* < 0.001).

Comparison of the two cell lines revealed that at 1 h, ZnPcSmix alone treated DLD-1 cells had significantly decreased H_2_O_2_ levels compared to similarly treated Caco-2 (*p* < 0.05), and at 24 h PDT treated DLD-1 cells had significantly decreased H_2_O_2_ levels compared to PDT treated CaCo-2 cells (*p* < 0.01).

### 2.2. Mitochondrial Membrane Potential

**Figure 2:**
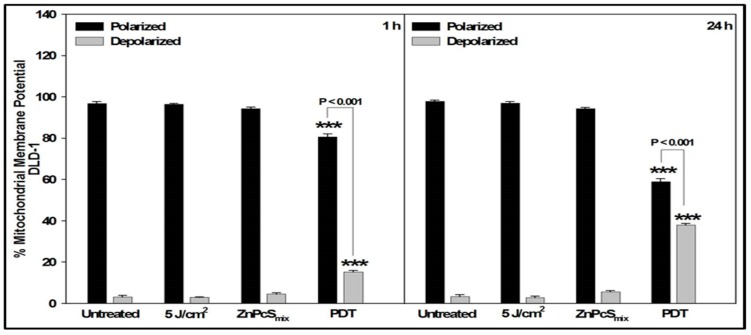
Loss of mitochondrial membrane potential in DLD-1 cells was analyzed 1 or 24 h post-treatment by JC-1 staining using flow cytometry. Significant differences as compared to untreated cells as shown as *** *p* < 0.001. After 1 h incubation there was an increase in depolarized PDT cells compared to all control cells, which increased significantly after 24 h.

JC-1 stain was used to assess mitochondrial membrane potential (∆Ψ). Cells treated with Actinomycin D were used as positive controls for apoptosis. The JC-1 flow cytometric dot plot in non-treated and treated cells were shown in the Supplementary Figures S1 and S2. After 1 h incubation, untreated, irradiated (5 J/cm^2^), ZnPcSmix alone and PDT treated DLD-1 cells had a significant percentage of cells that had polarized mitochondria compared to those that were depolarized (*p* < 0.001). However, the percentage of polarized PDT treated DLD-1 cells was significantly decreased as compared to the percentage of polarized cells in untreated, irradiated and ZnPcSmix treated control cells (*p* < 0.001); and the number of depolarized cells increased (*p* < 0.001) (Figure 2).

After 24 h, PDT treated DLD-1 cells still showed a significant decrease in polarized cells (*p* < 0.001) and increase in depolarized cells (*p* < 0.001) (Figure 1). The percentage of polarized PDT treated DLD-1 cells significantly decreased at 24 h compared to 1 h (*p* < 0.001), while depolarized cells significantly increased (*p* < 0.001).

**Figure 3:**
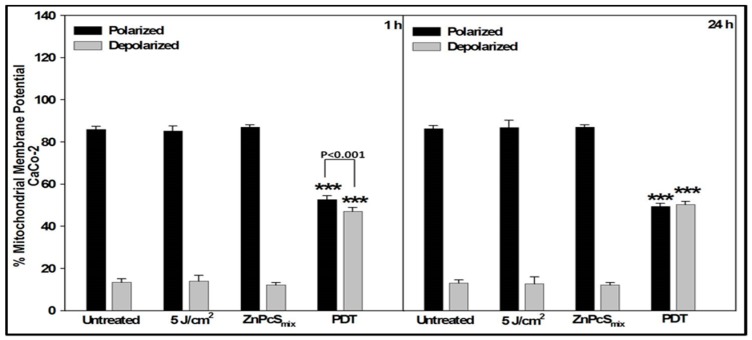
Loss of mitochondrial membrane potential in Caco-2 cells was analyzed 1 or 24 h post-treatment by JC-1 staining using flow cytometry. Significant differences as compared to untreated cells as shown as *** *p* < 0.001. At both incubation periods, there was a significant loss of mitochondrial membrane potential in PDT treated cells.

At 1 h, the percentage of polarized Caco-2 cells in all the groups was significantly increased as compared to depolarized cells (*p* < 0.001). The percentage of depolarized PDT treated Caco-2 cells was significantly increased as compared to all the other groups (*p* < 0.001). After 24 h, PDT treated Caco-2 cells still showed a significant decrease in polarized cells (*p* < 0.001) and increase in depolarized cells (*p* < 0.001) (Figure 3).

Comparison of the two cell lines showed that the percentage of polarized mitochondria in DLD-1 cells was significantly increased compared to that of similarly treated Caco-2 cells, at both 1 and 24 h (*p* < 0.01); likewise for the percentage of depolarized mitochondria decreased in DLD-1 cells (*p* < 0.01).

### 2.3. Cell Death Pathway (Apoptosis Array)

RNA was isolated in technical experimental triplicates and converted to cDNA prior to gene analysis. Quantitative real time RT-PCR was performed to analyze the up- or down-regulation of cell death genes 24 h post-PDT. DLD-1 cells had three significantly up-regulated genes and 20 significantly down-regulated genes (Table 1). Caco-2 cells had 16 significantly up-regulated genes and 22 significantly down-regulated genes (Table 2).

## 3. Discussion

PDT has received considerable attention as a potential therapeutic modality for cancer due to its attractive features of being a minimally invasive therapy. Although colorectal cancer is among the most lethal cancers in the world, data regarding the use of PDT as an alleviation therapy in colorectal cancer is limited, and no recent studies have shown the progress in PDT or development of PSs for use in the treatment of colorectal cancer. In this study, a transition metal (Zn) conjugated to a Pc structure with mixed sulfonation groups (ZnPcSmix) was used as a PS. Few studies have been conducted using the latter PS; therefore, a considerable amount of work still needs to be done in this regard. In previous work by Manoto et al. (2012), it was shown that DLD-1 colorectal cancer cells were sensitive to ZnPcSmix alone and ZnPcSmix-PDT. The morphological features of DLD-1 and Caco-2 control cells (0; 5 J/cm^2^ or ZnPcSmix) remained unchanged although the cells that received PDT treatment developed apoptotic features including detaching from the culture dish surface, cellular shrinking and cytoplasmic fragmentation [12,13].

In our previous work, it was determined that the preferential localization site of the ZnPcSmix was lysosomes and partially in the mitochondria [14]. This suggests that ZnPcSmix has chemical properties such as amphiphilicity and cationic moieties that target both lysosomes and mitochondria [4]. Since ZnPcSmix preferentially localizes in the lysosomes and mitochondria, it was important to determine if there was a leakage of the components located in lysosomes and mitochondria as a result of membrane destabilization. If high concentrations of ROS formed around the lysosomes, subsequent higher levels of H_2_O_2_ could diffuse into the lysosomes and react with intra-lysosomal metallo-proteins present and that have degraded as a result of low pH. This results in the iron reduction and the formation of hydroxyl radicals that promote lipid peroxidation, causing leakage of lysosomal contents [15]. Although ZnPcSmix alone also induced significant leakage of the aspartic enzyme after 1 h incubation in DLD-1 cells and 24 h in Caco-2 cells in comparison to untreated control cells, the leakage decreased. Leakage may be a result of prolonged incubation with the PS leading to osmotic stress and membrane permeabilization [16].

Significantly increased cytosolic H_2_O_2_ could be due to changes in the biophysical membranes of lysosomes and mitochondria caused by ROS or photodamage, which subsequently translates into changes in the permeability rate of lipophilic compounds such as H_2_O_2_. The significant presence of H_2_O_2_ levels in the cytosol of PDT treated cells was an indication that there was a rapid accumulation of the compound as compared to other control cells. Therefore, the detected H_2_O_2_ in the PDT treated colorectal cancer cells is linked with ROS production.

Loss of MMP is recognized as a cell death signal as it signifies loss of normal mitochondrial function, which as a result mitochondrial contents leak into the cytosolic space and trigger other elements to proceed with cell death. The JC-1 cationic dye demonstrates potential-dependent accumulation in the mitochondria denoting mitochondrial depolarization and subsequently cell death [17]. The majority of control cells had a high percentage of polarized mitochondria as opposed to PDT treated cells that showed a decrease in mitochondrial polarity. These findings also corroborate the presence of leaked cytochrome C into the cytosol as destabilization of mitochondrial function is proven by leakage of mitochondrial contents, such as cytochrome C, into the cytoplasm which subsequently activates apoptosis inducing agents [18].

Cancer cells are known to manipulate various cell mechanisms as a way of escaping cell death. They commonly have mutations in the pro-apoptotic proteins, over-expression of anti-apoptotic proteins and counter lysosomal leakage of hydrolases that can initiate cell death signals [19]. Gene expression in both cells lines proved to be different, which can be based on the fact that DLD-1 and Caco-2 cells are at two different Duke’s stages, Duke’s C and Duke’s B stage respectively. In DLD-1 PDT treated cells, there was an up-regulated expression of *NAIP* (Neuronal Apoptosis Inducing Factor), *NFκB1* (Nuclear Factor kappa B-1) and *TNFRSF21* (Tumour Necrosis Factor Receptor Superfamily 21). NAIP acts by inhibition of autocleavage of pro-caspase 9 and cleavage of caspase 3 by caspase 9 [20,21]; but it is known to induce caspase 1 in immune response regulation by cleaving IL-1 beta (IL-1β) and IL-18, in this manner the inflammatory response is initiated and cell death pursues [22]. The latter is defined as pyroptosis PCD, but it involves pro-inflammatory cytokines [23,24]. Additionally, *IL10*, which codes for an anti-inflammatory cytokine, was down-regulated which supports the inflammatory process the cell may be undergoing as its up-regulation is known to block NFkB-1 and may lead to survival [25].

In advanced prostate cancer, elevated NAIP is an early event, and its cytoprotective effects are associated with the NFkB-1 transcription factor signaling pathway [26,27]. This is also supported in this study as *NFkB1* was up-regulated. On the other hand, apoptosis inducing death receptor belonging to the TNF receptor family, *TNFRSF21*, also known as DR6, was up-regulated. The TNF superfamily proteins can also play a role in apoptosis, survival and proliferation [28]. The suggested mechanism may be through a unique mitochondrial pathway by interacting with Bax (apoptosis promoter) [29,30]. Interestingly, DR6 is known to induce NFkB-1 activation and its cognate ligand has not been identified and this happens to corroborate with this study as there was no up-regulated TNF ligand [28,31,32].

TNFRI Associated Death Domain (*TRADD*) that is thought to play a role in DR6 mediated apoptosis was down-regulated), and pro-apoptotic *BID* (BH3 interacting death domain death agonist) and *BAK1* as well as *TNF*. Cleavage and activation of Bid results in tBid, that subsequently translocates to the mitochondria and induces the release of proapoptotic mediators such as cytochrome C and apoptosis inducing factor (AIF). The release of the latter proapoptotic mediators is mediated by Bak or Bax because Bid facilitates the insertion of Bak or Bax into the mitochondrial membrane [33,34,35]. In this study, it is interesting that there was cytosolic cytochrome C, although *BAK1* was down-regulated, which possibly suggests that the apoptosis induction was mitochondria-independent. *MCL1* (myeloid cell leukemia), *BCL2L2* (BCL2-like2) and *BCL2L10* (BCL2-like10) code for anti-apoptotic proteins. They were found to be down-regulated, denoting they failed to inhibit apoptosis; although it is puzzling because they are known to antagonize Bax and Bak, which may be expected to be up-regulated. However, it has been hypothesized that they can play other roles that are not related to regulation of MMP [36,37,38]. Nonetheless, according to Beverly et al. (2013) and Merino et al. (2012), cancer cells evade apoptosis by relying on up-regulation of Bcl-2 like proteins for survival, therefore it is a good that ZnPcSmix mediated PDT has an ability to down-regulate *BCL2L2* and *BCL2L10* [36,39].

**Figure 4:**
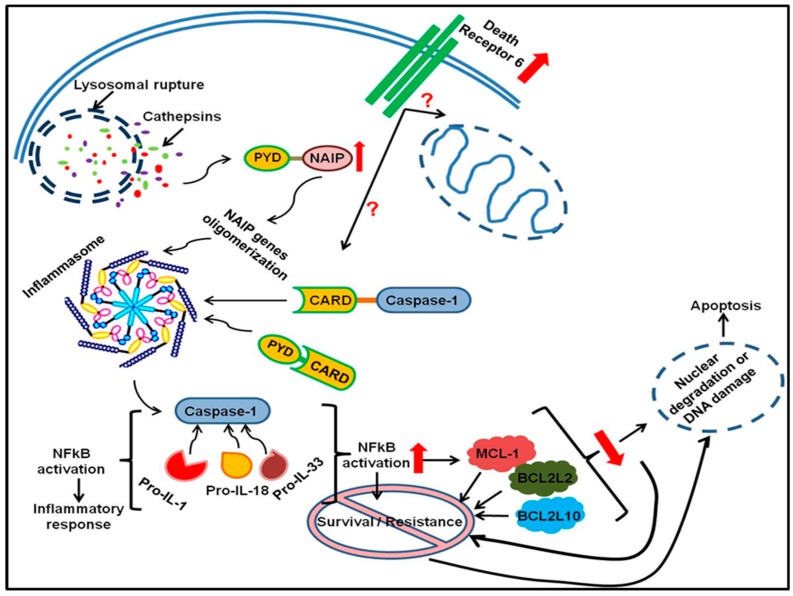
Up- and down-regulated genes in DLD-1 cells summarizing the possible mode of cell death induced by ZnPcSmix-PDT. Red arrows show either up- or down-regulated genes. Up-regulated death receptor 6 (*DR6*) may signal mitochondrial-induced cell death or induction of inflammation. Rupturing of lysosomes releases acidic lysosomal contents such as cathepsins, consequently followed by activation of NAIP. NAIP and PYCARD play a role in preceding inflammatory actions via NFkB-1 activation. However, since *NFkB1* was up-regulated, and *MCL1*, *BCL2L2* and *BCL2L10* were down-regulated, evasion of apoptosis was unsuccessful thus leading to DNA damage and apoptosis.

*CD70* and *CD27* were down-regulated, and CD70 is a CD27 ligand. CD27 can oligomerize with the adaptor proteins TRAF-2 and TRAF-5, and signal through NFkB-1 via NFkB-1 inducing kinase (NIK) that is associated with anti-apoptotic events. Signaling through TRAF is associated with survival, migration and differentiation [40,41,42,43]. Also, *CD40* and its ligand *CD40LG* were down-regulated, and they are known to play a role in cellular proliferation [28]. Therefore, the down-regulation seen in this study signifies that cell survival and proliferation was discouraged and also proves that apoptosis prevailed. The postulated mode of cell death in DLD-1 cells is depicted in Figure 4.

PDT induces apoptosis in advanced colorectal cancer cells (DLD-1) through DR6 and this finding has never been reported before as far as PDT-mediated cell death is concerned. Although it is not known what cell death mechanism is prompted by DR6, it can be postulated that the prevailing mode of cell death in DLD-1 cells is extrinsic as supported by DR6 expression which may be a unique mitochondrial pathway, as suggested by Zeng et al. (2012) considering the localization site of ZnPcSmix [29].

In Caco-2 cells, there was an up-regulation of *CASP10* and *TNF*, while *CASP8* was down-regulated. Caspase 8 is closely related to caspase 10, as they follow the same pathway of apoptosis induction. Caspase 10 is known to be up-regulated upon drug-induced DNA damage, which subsequently results in apoptosis by forming a DISC [26,44]. Caspase 2 has features of initiator (e.g., caspase 8) and executor (e.g., caspase 3) caspases, but its role has not been fully established. Apparently, the function of caspase-2 together with caspase-8 is to cleave Bid to active tBid, which subsequently leads to the induction of MMP and the downstream consequences of this event.

*CASP8* was down-regulated, suggesting that the extrinsic pathway may have not been the putative mode of cell death, which could be explained by the down-regulated apoptosis-inducing *FASLG* and *CD40LG* ligands. These ligands belong to the TNF superfamily and their cognitive receptors are FAS (CD95), CD40 and TNFR1 or TNFR2, respectively [28,45]. In cancer cells, over-expression of FASLG has been associated with increased risk of cancer progression while that of CD40L has been associated with proliferation and tumor progression, thereby promoting resistance by strengthening immunity [46,47,48,49,50]. Therefore, the down-regulation of *FASLG* and *CD40LG* in this study may be indicative of lower chances of recurrence post-PDT and therapeutic effects of ZnPcSmix-mediated PDT.

*TNFRSF9* (CD137), *TNFSF8* (CD30) and *TNFRSF10A* (DR4) genes belonging to the TNF receptor family were up-regulated. According to Kwon (2012) and Chakrabarty et al. (2003), TNFRSF9 and TNFSF8 play a role in the inflammatory response or regulation and facilitation of pathogen clearance, and thus contribute to the rapid resolution of the inflammatory process [51,52]. TNFSF8 signaling can favor apoptosis, proliferation or differentiation as a means of regulating the inflammatory response [52]. TNFSF8 triggers cell death indirectly by stimulating the production of TNF and sensitizing cells to apoptosis through TNFRI, which may be the case in this study, since *TNF* was up-regulated as well as TNF related receptors [53].

*NAIP* and *PYCARD* (Pyrin Domain, PYD and Caspase-Recruitment Domain, CARD) genes were significantly up-regulated in Caco-2 cells. PYCARD belongs to the death domain-fold superfamily that mediates assembly of large signaling complexes in the inflammatory and apoptotic signaling pathways via the activation of caspase and PYD is involved in proteolytic cleavage of pleiotropic inflammatory cytokines such as IL-1β and IL-18, while CARD is involved in Apaf-1 and caspase 9 protein interactions [54,55,56]. A recent study conducted by Hong et al. (2013) in colorectal cancer cell lines showed that PYCARD was capable of inducing both apoptotic and necrotic cell death [57]. Nonetheless, Hong et al. (2013) concluded that PYCARD expression clearly promotes colorectal cancer cell death in response to genotoxic stress, thus being suggestive of a tumor-suppressive function. Therefore, up-regulation of *PYCARD* in PDT treated Caco-2 cells clearly demonstrates the ability of ZnPcSmix to induce cell death in cancer cells.

In this study, two growth promoting genes (*AKT1* and *IGF1R*) were down-regulated post-PDT treatment of Caco-2 cells. In breast cancer, the up-regulation of *IGF1R* and the activation of its downstream signaling molecules have been linked to an inhibition of apoptosis, disease progression, increased resistance to cytotoxic chemo-therapeutic drugs or radiotherapy, and to poor prognosis [58]. Therefore, it is remarkable that *IGF1R* and *AKT1* were down-regulated 24 h post-PDT, as this translates into there being less chance of survival and warrants PDT-induced cell death. Paradoxically, genes encoding for pro-apoptotic proteins, *CIDEA* (Cell death Inducing DNA fragmentation factor Effector A), *BAX* and *BAD* were down-regulated, while *BAK1* and *BIK* were up-regulated. The latter genes share structural homology and facilitate downstream apoptosis signaling. Activation of Bax or Bak by Bik is crucial for mediation of the MMP, leading to the consequent release of apoptogenic factors from the mitochondria such as cytochrome C and Smac/DIABLO into the cytosol [59,60,61].

**Figure 5:**
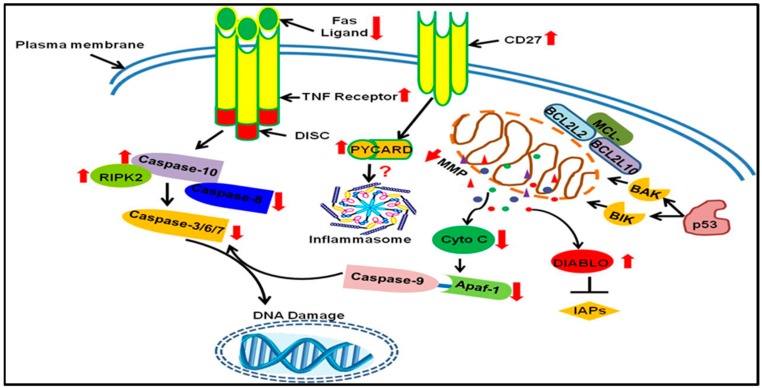
Possible mode of cell death pathway in Caco-2 cells. Red arrows show either up- or down-regulated genes. Tumor necrosis factor receptor (*TNFR*) was up-regulated, signaling its proactive role in the receptor induced pathway and involvement of Caspases that precede apoptotic nuclear damage. *TP53* (p53) activates pro-apoptotic genes (*BAK* and *BIK*), resulting in decreased mitochondrial membrane potential (MMP) and subsequent release of mitochondrial apoptotic contents such as cytochrome C (Cyto C) and direct IAP binding protein with low pI (*DIABLO*). DIABLO deactivates inhibitors of apoptosis proteins (IAPs) in order to potentiate apoptosis. Up-regulated *CD27* and *PYCARD* are suggestive of an inflammatory response; however, *NF_K_β1* was neither up- nor down-regulated in Caco-2 cells, hence the uncertainty (?) regarding the involvement of inflammasomes.

*DIABLO* was up-regulated in Caco-2 cells, with a fold change of 7.82, suggesting that although *BAX* or *BAD* was not up-regulated, activation of Bak and Bik was enough to mediate MMP as supported by the JC-1 results, and release of cytochrome C into the cytosol. This is also supported by the down-regulation of the *BIRC5* gene which is an inhibitor of apoptosis, because up-regulated expression of BIRC5 is known to bind and inactivate DIABLO and subsequently result in evasion of apoptosis; thus, its down-regulation proves that there was apoptosis. Bik also indirectly drives apoptosis by sensitizing or reducing free anti-apoptotic Bcl-2 family members which could have been the case in this study since *MCL1*, *BCL2L10* and *BCL2L11* were up-regulated [62,63]. The proposed apoptotic activation in Caco-2 cells is summarized in Figure 5.

In both cell lines, in up-regulated genes, there was a combination of pro- and anti-apoptotic genes that were significantly expressed. Gene expression results showed that more tumorigenic cells (DLD-1) went through apoptosis; however, they exhibited great chances of resistance and recurrence. Meanwhile, less tumorigenic Caco-2 cells showed better response to PDT, thus being suggestive of a better prognosis post-PDT treatment. In conclusion, ZnPcSmix-mediated PDT bears great potential as a PS for colon cancer treatment, especially because it targets two potent organelles. In conclusion, ZnPcSmix is a good PS, as it provides the best of both worlds, because it is localized in two magnanimous organelles that play a pivotal role in cell death machinery. Our results presented in this paper suggest lysosomal initiation of apoptotic cell death in response to PDT. In turn, delayed mitochondrial cytochrome C leakage as induced by the proteolytic enzyme cathepsin D as well as decreased pH resulting from the lysosomes support our findings. However, further research including western blot analysis to confirm and support expression of apoptotic proteins should be conducted to elucidate the exact cell death mechanisms.

## 4. Materials and Methods

### 4.1. Cell Culture

This research study was approved by the University of Johannesburg, Faculty of Health Sciences Academic Ethics Committee (AEC81/2009). Two human colorectal adenocarcinoma cell lines, each in a different stage of cancer, were utilized. Caco-2 (ATCC^®^ HTB-37™) cells are in Dukes stage B, meaning the cancer has grown through the muscle layer of the bowel, while DLD-1 cells (ATCC^®^ CCL-221™) are in Dukes stage C meaning the cancer has metastasized to at least one lymph node in the area close to the bowel. Cells were grown under conventional conditions as previously described [14]. For experiments, 5 × 10^4^ cells in 3 mL complete media were seeded into 3.4 cm diameter culture plates and incubated overnight to allow for attachment. Cells were randomly divided into four groups; cells that neither received PS nor irradiation (untreated), cells that received irradiation alone (5 J/cm^2^), cells that received PS alone (ZnPSmix), and cells that received both PS and irradiation (PDT).

### 4.2. PDT Experiments

The PS that was used in this study was a ZnPcSmix with a peak absorbance at 680 nm and contained various sulfo groups. The PS was designed and characterized by the Department of Chemistry, Rhodes University, South Africa [64]. Following overnight cellular attachment, cells were rinsed with Hank’s Balanced Salt Solution (HBSS) and media replaced with 1 mL fresh complete media and left for 30 min. ZnPcSmix at a concentration of 20 µM was added to the relevant groups and cells incubated for 24 h. Cells were rinsed twice with HBSS and the PDT and laser irradiation alone (5 J/cm^2^) groups were irradiated in the dark from above using a 680 nm continuous diode laser (power output of 42 mW, spot size of 9.1 cm^2^, power density of 4.6 mW/cm^2^, irradiation time 1080 s). Cells were irradiated with a fluence of 5 J/cm^2^, as this was determined to be the optimum from dose response studies [12]. Biological responses were evaluated after a further incubation for 1 or 24 h in fresh, PS-free media.

### 4.3. Cytosolic Acidification—Hydrogen Peroxide (H_2_O_2_) Production

Hydrogen peroxide (H_2_O_2_) levels were determined using a quantitative fluorometric cell-based assay kit (Cayman Chemical, 600050, Michigan, MI, USA) according to the manufacturer details. In brief, cells were centrifuged at 400× *g* for 5 min and 80 µL of standard (8-point serial dilution) or cell supernatant were transferred in duplicate into black-walled microplates. Ten microliters (10 µL) of assay buffer was added followed by 10 µL of enzyme reaction solution and incubation at room temperature for 30 min with gentle rocking. Fluorescence intensity was read using the Victor^3^ (Perkin-Elmer, Johannesburg, South Africa) microplate reader at 530_Ex_/590_Em_.

### 4.4. Mitochondrial Destabilization

Mitochondrial membrane potential (∆Ψ) was detected through mitochondrial staining using JC-1 (5,5′,6,6′-tetrachloro-1,1′,3,3′-tetraethylbenzimidazolcarbocyanine iodide) according to the manufacturer’s details (BD Biosciences, 551302, Sandton, South Africa). JC-1 is rapidly taken up by polarized mitochondria in normal healthy cells, resulting in the creation of JC-1 aggregates that show a red spectral shift with higher levels of red fluorescence emission (measured in the red, FL-2, channel). JC-1 does not accumulate in depolarized mitochondria (as occurs in apoptotic cells) and remains as monomers in the cytoplasm. These monomers do not display the red spectral shift, and therefore have lowered fluorescence in the FL-2 channel. JC-1 aggregates and monomers exhibit fluorescence in the green end of the spectrum (measured in the green, FL-1 channel). Healthy cells will have a polarized mitochondrial membrane potential, and hence show JC-1 fluorescence in both the FL-1 and FL-2 channels, while cells with a depolarized mitochondrial membrane potential will display JC-1 fluorescence in the FL-1 channel and lack fluorescence in the FL-2 channel.

Detached cells were re-suspended in 1 mL HBSS and centrifuged at 400× *g* for 5 min. The supernatant was discarded and cells re-suspended in 0.5 mL freshly prepared JC-1 working solution and incubated in a CO_2_ incubator at 37 °C for 10 min. Cells were washed in 1× Assay Buffer and centrifuged at 400× *g* for 5 min. The supernatant was discarded, cells washed and centrifuged as before and re-suspended in 0.5 mL 1× Assay buffer and analyzed by flow cytometry on the FACSCAria (BD Biosciences).

### 4.5. Expression of Apoptotic Genes

The RNeasy mini QIAcube RNA isolation kit (Qiagen, 74116, Randburg, South Africa) was used in the QIAcube (Qiagen) as per the manufacturer instructions and previously described [65]. Total RNA was isolated from cells untreated control and PDT treated cells 24 h post-PDT. Total isolated RNA was quantified on the Qubit™ fluorometer (Invitrogen, Thermo Fisher Scientific, Johannesburg, South Africa) using the Quant-iT™ RNA Assay kit (Invitrogen^TM^ Q32852). Purity was determined spectrophotometrically in 10 mM Tris chloride (Tris-Cl), pH 7.5 (1:100 dilution) at A260 nm/A280 nm. cDNA was synthesized from 1 μg total RNA using the QuantiTect Reverse Transcription kit (Qiagen, 205311) as per the manufacturer instructions and previously described [65]. cDNA purity was determined (A260/A280). cDNA samples were stored at −20 °C. Real-time qPCR was performed on the Stratagene Mx3000p using the human apoptosis RT^2^ Profiler™ PCR Array (Qiagen 330231) to profile the expression of 84 key genes involved in apoptosis (Table 3). qPCR was carried out according to the manufacturer instructions and previously described [65]. The thermal cycler was set to run 10 min at 95 °C for 1 cycle and 15 s at 95 °C and 1 min at 60 °C for 40 cycles, after which a melt/dissociation curve was performed (95 °C for 1 min, 55 °C for 30 s and 55 °C to 95 °C at 2 °C per minute). Cycle threshold (*C*t) values were imported into an Excel spreadsheet (PAHS-012Z Available from the Qiagen website: http://www.qiagen.com/) which normalized results against an average of 5 housekeeping genes (*ACTB*, *B2M*, *GAPDH*, *HPRTI*, and *RPLPO*). Relative gene expression (ΔΔ*C*t) and fold change (2^−ΔΔ*C*t^) as compared to untreated controls were also calculated. A fold change of >1 was reported as fold up-regulation and a f old change <1 was reported as fold down-regulation.

### 4.6. Statistical Analysis

Biochemical experiments were repeated four times (*n* = 4), while PCR arrays were repeated three times (*n* = 3). Biochemical assays were done in duplicate, of which the average of the two was used. Statistical analysis was performed using SigmaPlot version 8.0 (Systat Software, San Jose, CA, USA). Differences between groups was determined using the one-tailed Student’s *t*-test and One-Way Analysis of Variance (ANOVA). Data is plotted using standard error bars (SEM) and statistical differences are shown in graphs as (*) *p* < 0.05, (**) *p* < 0.01 and (***) *p* < 0.001.

## Figures and Tables

**Table 1:** Gene expression in DLD-1 cells. Up-regulated genes had a fold change (2^(−∆∆Ct)^) above 1, while down-regulated genes had a 2^(−∆∆Ct)^ below 1. Only statistically significant genes are shown.

Gene Symbol	Gene Name	*p* Value	Fold-Change 2^(−∆∆Ct)^	Up (↑)/Down (↓) Regulation	Function
*ABL1*	ABL proto-oncogene 1, non-receptor tyrosine kinase	<0.05	0.29	↓	Positive Regulation of Apoptosis
*BAK1*	BCL2 antagonist/killer 1	<0.05	0.11	↓	Pro-Apoptotic/Positive Regulation of Apoptosis
*BCL2L10*	BCL2 like 10	<0.001	0.19	↓	Anti-Apoptotic
*BCL2L2*	BCL2 like 2	<0.05	0.07	↓	Anti-Apoptotic
*BID*	BH3 interacting domain death agonist	<0.05	0.29	↓	Pro-Apoptotic/Positive Regulation of Apoptosis
*CASP14*	Caspase 14	<0.001	0.19	↓	Pro-Apoptotic/Positive Regulation of Apoptosis/Caspase
*CD27*	CD27 molecule	<0.001	0.22	↓	Pro-Apoptotic/Anti-Apoptotic/Negative Regulation of Apoptosis/Caspase inhibitor
*CD40*	CD40 molecule	<0.001	0.22	↓	Positive Regulation of Apoptosis
*CD40LG*	CD40 ligand	<0.05	0.25	↓	Anti-Apoptotic
*CD70*	CD70 molecule	<0.01	0.28	↓	Pro-Apoptotic/Positive Regulation of Apoptosis
*CIDEA*	Cell death inducing DFFA like effector a	<0.01	0.17	↓	Negative Regulation of Apoptosis/DNA Damage & Repair
*IL10*	Interleukin 10	<0.05	0.24	↓	Anti-Apoptotic
*LTBR*	Lymphotoxin beta receptor	<0.001	0.10	↓	Positive Regulation of Apoptosis
*MCL1*	MCL1 apoptosis regulator, BCL2 family member	<0.01	0.10	↓	Negative Regulation of Apoptosis
*NAIP*	NLR family apoptosis inhibitory protein	<0.001	5.69	↑	Anti-Apoptotic/Negative Regulation of Apoptosis
*NFkB1*	Nuclear factor kappa B subunit 1	<0.01	3.09	↑	Anti-Apoptotic
*PYCARD*	PYD and CARD domain containing	<0.01	0.28	↓	Pro-Apoptotic/Positive Regulation of Apoptosis/Caspase Regulator
*RIPK2*	Receptor interacting serine/threonine kinase 2	<0.05	0.23	↓	Anti-Apoptotic/Positive Regulation of Apoptosis
*TNF*	Tumor necrosis factor	<0.05	0.70	↓	Death Domain Receptor/Anti-Apoptotic/Positive Regulation of Apoptosis
*TNFRSF21*	TNF receptor superfamily member 21	<0.01	284.18	↑	Death Domain Receptor
*TNFSF8*	TNF superfamily member 8	<0.001	0.18	↓	Pro-Apoptotic/Positive Regulation of Apoptosis
*TP73*	Tumor protein p73	<0.01	0.20	↓	DNA Damage & Repair/Negative Regulation of Apoptosis
*TRADD*	TNFRSF1A associated via death domain	<0.05	0.09	↓	Pro-Apoptotic/Positive Regulation of Apoptosis/Death Domain Receptor

**Table 2:** Gene expression in Caco-2 cells 24 h post-PDT. Up-regulated genes had a fold change (2^(−∆∆Ct)^) above 1, while down-regulated genes had a 2^(−∆∆Ct)^ below 1. Only statistically significant genes are shown.

Gene Symbol	Gene Name	*p* Value	Fold-Change 2^(−∆∆Ct)^	Up (↑)/Down (↓) Regulation	Function
*AKT1*	AKT serine/threonine kinase 1	<0.001	0.11	↓	Anti-Apoptotic/Positive Regulation of Apoptosis
*APAF1*	Apoptotic peptidase activating factor 1	<0.01	0.21	↓	Caspase Activator
*BAD*	BCL2 associated agonist of cell death	<0.01	0.29	↓	Pro-Apoptotic/Positive Regulation of Apoptosis
*BAK1*	BCL2 antagonist/killer 1	<0.05	3.07	↑	Pro-Apoptotic/Positive Regulation of Apoptosis
*BAX*	BCL2 associated X, apoptosis regulator	<0.001	0.24	↓	Pro-Apoptotic/Anti-Apoptotic/Positive Regulation of Apoptosis/Caspase Activator
*BCL2*	BCL2 apoptosis regulator	<0.001	0.22	↓	Anti-Apoptotic/Negative Regulation of Apoptosis
*BCL2L1*	BCL2 like 1	<0.01	0.19	↓	Anti-Apoptotic
*BCL2L10*	BCL2 like 10	<0.05	2.27	↑	Anti-Apoptotic/Negative Regulation of Apoptosis
*BCL2L11*	BCL2 like 11	<0.05	2.27	↑	Pro-Apoptotic
*BIK*	BCL2 interacting killer	<0.05	2.27	↑	Pro-Apoptotic/Positive Regulation of Apoptosis
*BIRC5*	Baculoviral IAP repeat containing 5	<0.01	0.23	↓	Anti-Apoptotic
*BNIP2*	BCL2 interacting protein 2	<0.05	0.38	↓	Anti-Apoptotic/Negative Regulation of Apoptosis
*CASP10*	Caspase 10	<0.05	2.27	↑	Positive Regulation of Apoptosis/Caspase
*CASP14*	Caspase 14	<0.05	2.27	↑	Pro-Apoptotic/Positive Regulation of Apoptosis/Caspase
*CASP2*	Caspase 2	<0.001	0.19	↓	Pro-Apoptotic/Positive Regulation of Apoptosis/Caspase
*CASP6*	Caspase 6	<0.001	0.53	↓	Pro-Apoptotic/Positive Regulation of Apoptosis/Caspase
*CASP8*	Caspase 8	<0.01	0.34	↓	Pro-Apoptotic/Positive Regulation of Apoptosis/Caspase
*CD27*	CD27 molecule	<0.05	1.99	↑	Pro-Apoptotic/Anti-Apoptotic/Negative Regulation of Apoptosis/Caspase inhibitor
*CD40LG*	CD40 ligand	<0.01	0.40	↓	Anti-Apoptotic
*CIDEA*	Cell death inducing DFFA like effector a	<0.05	0.20	↓	Negative Regulation of Apoptosis/DNA Damage & Repair
*CYCS*	Cytochrome c, somatic	<0.01	0.34	↓	Pro-Apoptotic
*DIABLO*	Diablo IAP-binding mitochondrial protein	<0.001	7.82	↑	Pro-Apoptotic
*FADD*	Fas associated via death domain	<0.05	0.18	↓	Positive Regulation of Apoptosis/Death Domain
*FASLG*	Fas ligand	<0.01	0.23	↓	Pro-Apoptotic/Positive Regulation of Apoptosis
*GADD45A*	Growth arrest and DNA damage inducible alpha	<0.03	0.58	↓	Pro-Apoptotic
*IGF1R*	Insulin like growth factor 1 receptor	<0.001	0.13	↓	Anti-Apoptotic/Negative Regulation of Apoptosis
*LTBR*	Lymphotoxin beta receptor	<0.01	0.42	↓	Positive Regulation of Apoptosis
*MCL1*	MCL1 apoptosis regulator, BCL2 family member	<0.01	2.27	↑	Negative Regulation of Apoptosis
*NAIP*	NLR family apoptosis inhibitory protein	<0.05	2.27	↑	Anti-Apoptotic/Negative Regulation of Apoptosis
*PYCARD*	PYD and CARD domain containing	<0.05	2.73	↑	Pro-Apoptotic/Positive Regulation of Apoptosis/Caspase Regulator
*RIPK2*	Receptor interacting serine/threonine kinase 2	<0.01	2.28	↑	Anti-Apoptotic/Positive Regulation of Apoptosis
*TNF*	Tumor necrosis factor	<0.05	2.27	↑	Death Domain Receptor/Anti-Apoptotic/Positive Regulation of Apoptosis
*TNFRSF10A*	TNF receptor superfamily member 10a	<0.05	2.06	↑	Pro-Apoptotic/Positive Regulation of Apoptosis/Death Domain Receptor
*TNFRSF9*	TNF receptor superfamily member 9	<0.05	2.27	↑	Pro-Apoptotic/Positive Regulation of Apoptosis
*TNFSF8*	TNF superfamily member 8	<0.05	2.27	↑	Pro-Apoptotic/Positive Regulation of Apoptosis
*TP53*	Tumor protein p53	<0.01	0.38	↓	DNA Damage & Repair/Positive Regulation of Apoptosis/Caspase Activator
*TP73*	Tumor protein p73	<0.05	0.22	↓	DNA Damage & Repair/Negative Regulation of Apoptosis
*TRADD*	TNFRSF1A associated via death domain	<0.05	0.32	↓	Pro-Apoptotic/Positive Regulation of Apoptosis/Death Domain Receptor

**Table 3:** Functional genes that were analyzed simultaneously on the human apoptosis RT^2^ Profiler™ PCR array.

Functional Gene Grouping	Genes
Death Domain Receptors	*CRADD*, *FADD*, *TNF*, *TNFRSF10B* (*DR5*).
DNA Damage & Repair	*ANL1*, *CIDEA*, *CIDEB*, *TP53*, *TP73*.
Extracellular Apoptotic Signals	*CFLAR* (*CASPER*), *DAPK1*, *TNFRSF25* (*DR3*).
Other Pro-Apoptotic Genes	*BAD*, *BAK1*, *BAX*, *BCL10*, *BCL2L11*, *BID*, *BIK*, *BNIP3*, *BNIP3L*, *CASP1* (*ICE*), *CSP10* (*MCH4*), *CASP14*, *CASP2*, *CASP3*, *CASP4*, *CASP6*, *CASP8*, *CD27* (*TNFRSF7*), *CD70* (*TNFSF7*), *CYCS*, *DFFA*, *DIABLO* (*SMAC*), *FAS* (*TNFRSF6*), *FASLG* (*TNFSF6*), *GADD45A*, *HRK*, *LTA* (*TNFB*), *NOD1* (*CARD4*), *PYCARD* (*TMS1/ASC*), *TNFRSF10A*, *TNFRSF9*, *TNFSF10* (*TRAIL*), *TNFSF8. TP53BP2*, *TRADD*, *TRAF3*.
Anti-Apoptotic	*AKT1*, *BAG1*, *BAG3*, *BAX*, *BCL2*, *BCL2A1* (*Bfl-1/A1*), *BCL2L1* (*BCL-X*), *BCL2L10*, *BCL2L2*, *BFAR*, *BIRC3* (*cIAP1*), *BIRC5*, *BIRC6*, *BNIP2*, *BNIP3*, *BNIP3L*, *BRAF*, *CD27* (*TNFRSF7*), *CD40LG* (*TNFSF5*), *CFLAR* (*CASPER*, *DAPK1*, *FAS* (*TNFRSF6*, *HRK*, *IGF1R*, *IL10*, *MCK1*, *NAIP* (*BIRC1*), *NFKB1*, *NOL3*, *RIPK2*, *TNF*, *XIAP* (*BIRC4*).
Regulation of Apoptosis	**Negative regulation:***BAG1*, *BAG3*, *BCL10*, *BCL2*, *BCL2A1* (*Bfl-1/A1*), *BCL2L1* (*BCL-X*), *BCL2L10*, *BCL2L2*, *BFAR*, *BIRC2* (*c-IAP2*), *BIRC3* (*c-IAP1*), *BIRC6*, *BNIP2*, *BNIP3*, *BNIP3L*, *BRAF*, *CASP3*, *CD27* (*TNFRSF7*), *CD40LG* (*TNFSF5*), *CFLAR* (*CASPER*), *CIDEA*, *DAPK1*, *DFFA*, *FAS* (*TNFRSF6*), *IGF1R*, *MCL1*, *NAIP* (*BIRC1*), *NOL3*, *TP53*, *TP73*, *XIAP* (*BIRC4*). **Positive regulation:** *ABL1*, *AKT1*, *BAD*, *BAK1*, *BAX*, *BCL2L11*, *BID*, *BIK*, *BNIP3*, *BNIP3L*, *CASP1* (*ICE*), *CASP10* (*MCH4*), *CASP14*, *CASP2*, *CASP4*, *CASP6*, *CASP8*, *CD40* (*TNFRSF5*), *CD70* (*TNFSF7*), *CIDEB*, *CRADD*, *FADD*, *FASLG* (*TNFSF6*), *HRK*, *LTA* (*TNFB*), *LTBR*, *NOD1* (*CARD4*), *PYCARD* (*TMSS1/ASC*), *RIPK2*, *TNF*, *TNFRSF10A*, *TNFRSF10B* (*DR5*), *TNFRSF25* (*DR3*), *TNFRSF9*, *TNFSF10* (*TRAIL*), *TNFSF8*, *TP53*, *TP53BP2*, *TRADD*, *TRAF2*, *TRAF3*.
Death Domain Receptors	*CRADD*, *DAPK1*, *FADD*, *TNFRSF10A*, *TNFRSF10B* (*DR5*), *TNFRSF11B*, *TNFRSF1A*, *TNFRSF1B*, *TNFRSF21*, *TNFRSF25* (*DR3*), *TRADD*.
Caspases and Regulators	**Caspases:***CASP1* (*ICE*), *CASP10* (*MCH4*), *CASP14*, *CASP2*, *CASP3*, *CASP4*, *CASP5*, *CASP6*, *CASP7*, *CASP8*, *CASP9*, *CFLAR* (*CASPER*), *CRADD*, *PYCARD* (*TMS1/ASC*). **Caspase activators:** *AIFM1* (*PDCD8*), *APAF1*, *BAX*, *BCL2L10*, *CASP1* (*ICE*), *CASP9*, *NOD* (*CARD4*), *PYCARD* (*TMS1/ASC*), *TNFRSF10A*, *TNFRSF10B* (*DR5*), *TP53*. **Caspase inhibitors:** *CD27* (*TNFRSF7*), *XIAP* (*BIRC4*).

## Supplementary Materials

Supplementary materials can be found at https://www.mdpi.com/1422-0067/20/13/3254/s1.
