# Supplementary material for: Genetic Aberrations Associated with Photodynamic Therapy in Colorectal Cancer Cells
Source: Int J Mol Sci. 2019 Jul 2;20(13):3254. doi: 10.3390/ijms20133254 (PMC6651415; doi:10.3390/ijms20133254)
Supplement: Supplementary file 1 [file ijms-20-03254-s001.pdf]

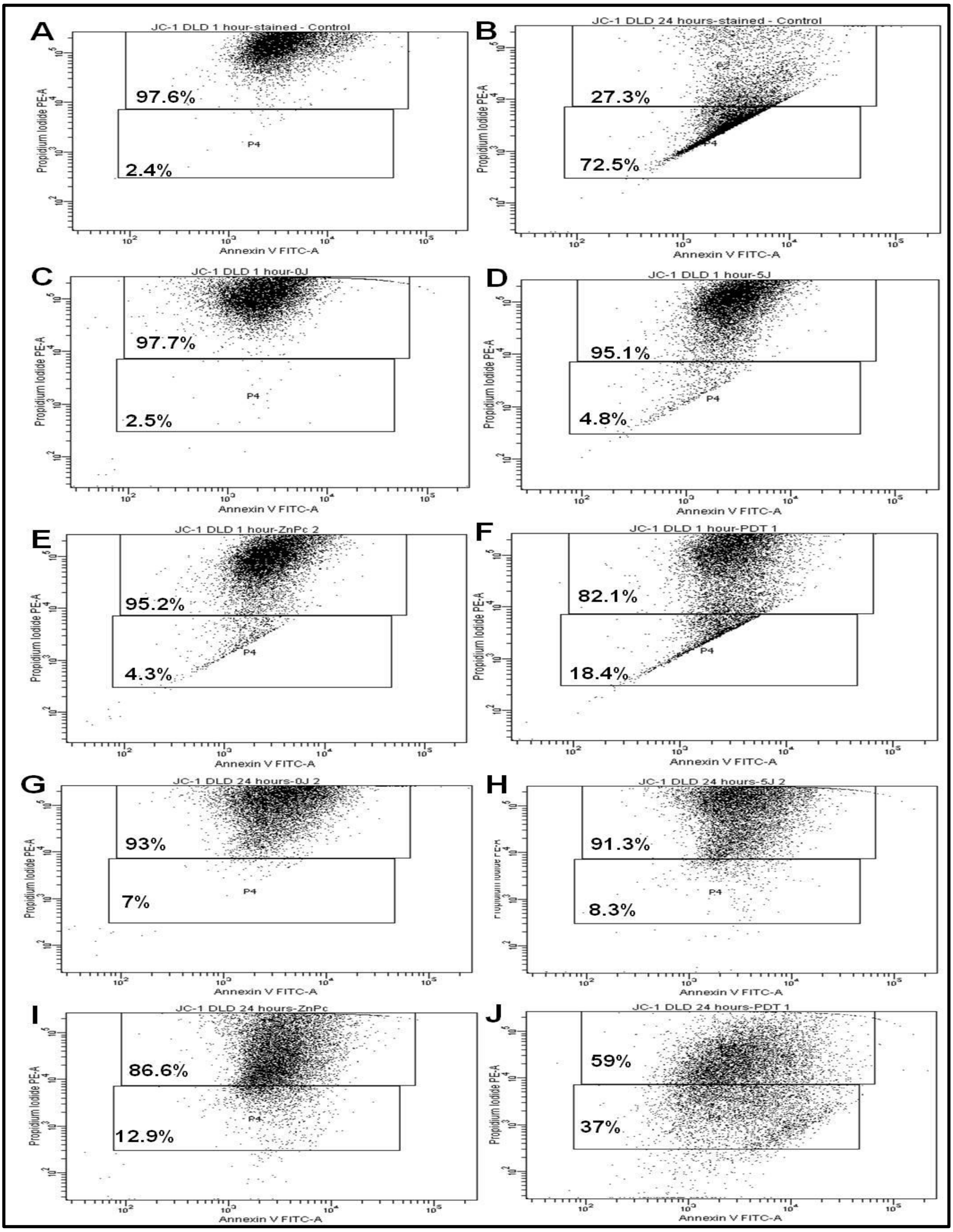

Figure S1 JC-1 flow cytometric dot plot. Dot plot A and B are negative and positive controls, respectively. Untreated, irradiated and $ZnPcS_{mix}$ treated DLD-1 cells consisted of a high percentage of polarized cells after 1 (C, D, E, respectively) and 24 h (G, H, I, respectively) incubation as seen on the top quadrant; whereas PDT treated DLD-1 cells had a significant portion of cells that had shifted to the green fluorescence (bottom quadrant) after 1 (F) and 24 h (J) incubation post-PDT, denoting cell death.

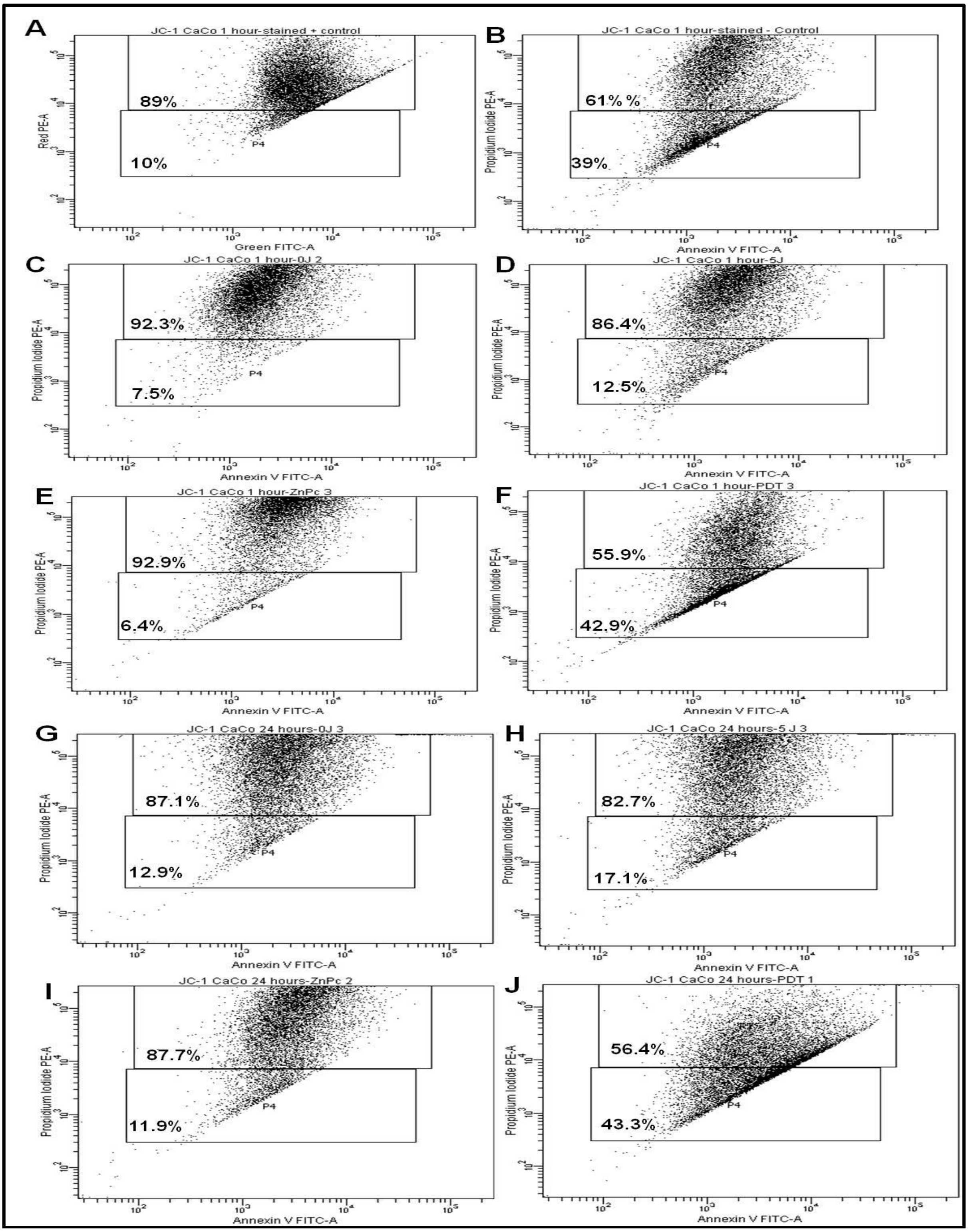

Figure S2 JC-1 flow cytometric dot plot. Dot plot A and B are positive and negative apoptotic controls, respectively. Untreated, irradiated and $ZnPcS_{mix}$ treated CaCo-2 cells consisted of a high percentage of polarized cells after 1 (C, D, E, respectively) and 24 h (G, H, I, respectively) incubation as seen on the top quadrant; whereas PDT treated CaCo-2 cells had a significant portion of cells that had shifted to the green fluorescence (bottom quadrant) after 1 (F) and 24 h (J) incubation post-PDT, denoting cell death.
